# Hepatitis B virus infection among pregnant mothers and children after the introduction of the universal vaccination program in Central Vietnam

**DOI:** 10.1038/s41598-021-87860-1

**Published:** 2021-04-21

**Authors:** Masami Miyakawa, Lay-Myint Yoshida, Hien-Anh Thi Nguyen, Kensuke Takahashi, Tho Huu Le, Michio Yasunami, Koya Ariyoshi, Duc-Anh Dang, Hiroyuki Moriuchi

**Affiliations:** 1 Department of Pediatrics, Nagasaki University Hospital, Nagasaki University, Nagasaki, 852-8102 Japan; 2 Department of Pediatric Infectious Diseases, Institute of Tropical Medicine, Nagasaki University, Nagasaki, 852-8523 Japan; 3 Graduate School of Biomedical Sciences, Nagasaki University, Nagasaki, 852-8102 Japan; 4 National Institute of Hygiene and Epidemiology, Hanoi, 100000 Vietnam; 5 Department of Clinical Medicine, Institute of Tropical Medicine, Nagasaki University, Nagasaki, 852-8523 Japan; 6 Khanh Hoa Provincial Health Service Department, Nha Trang, 650000 Vietnam

**Keywords:** Hepatitis B virus, Epidemiology

## Abstract

A birth cohort study was conducted in Khan Hoa Province, central Vietnam between 2009 and 2012 to determine the seroprevalence of hepatitis B virus (HBV) in pregnant women and their children, and associated risk factors. We enrolled 1987 pregnant women with their babies at the birth phase, and 12.6% (95% confidence interval [CI]: 11.1–14.0) of mothers were hepatitis B surface antigen (HBsAg)+. At 2-year follow-up phase, 1339 (67.4%) children were enrolled of whom 76.6% completed hepatitis B vaccines (HepB) and 1.9% (95% CI: 1.2–2.7) were HBsAg+. When mothers were hepatitis B e antigen (HBeAg)+, 28.3% of children have got infected even with complete HepB. HBV infection in mothers, hepatitis B surface antibody (anti-HBs antibody) below the seroprotective level in children, and mothers with pre-pregnancy low body mass index were associated with HBV infection in children. Meanwhile, HBV infection in children, older maternal age, no or incomplete doses of HepB, and boys were associated with anti-HBs antibody below the seroprotective level in children. Our birth cohort study determined a low rate of congenital HBV infection and associated risk factors in Vietnam, however further studies are needed to advance prevention including anti-viral therapy in pregnant women at high risk.

## Introduction

Hepatitis B virus (HBV) remains a major public health challenge in Asia. Most of the HBV disease burden results from infections acquired perinatally (80–90%) or during early childhood (30–50%)^1,2^.

Hepatitis B surface antigen (HBsAg)+, in particular, hepatitis B e antigen (HBeAg)+mother or mothers with high viral load of HBV are at a higher risk of mother-to-child transmission: 70–100% of children born to HBeAg+mothers and 5–30% of those born to HBeAg- mothers in Asia^1^.

A primary 3-dose hepatitis B vaccine (HepB3) immunization would induce the protective immunity to HBV in > 95% of healthy infants and children, and birth dose (HepB-BD) administered within 24 h of birth could reduce chronic infection rate^1^. The use of this cost-effective HepB vaccination in infants has considerably reduced the incidence of new chronic HBV infections among children under 5 years of age globally from 4.7% in the pre-vaccination era to 1.3% in 2015^3^.

Hepatitis B surface antibody (anti-HBs antibody) concentrations would rapidly decline within the first year and gradually thereafter^4^, while long-term immunologic memory and persistence of protection against HBV may last for years despite waning anti-HBs antibody levels^1,4–8^.

Vietnam is one of the highly HBV-endemic countries^1^ with 8.8–19.0% of HBsAg prevalence in adult population^9–13^ including 9.5% in pregnant women^10^. 16.4–38.2% of HBsAg+adults were HBeAg+^12–14^.

Vietnam introduced a nationwide HepB vaccination program in 2002. It was initially scheduled as HepB-BD plus 2 doses of monovalent HepB, which was revised into HepB-BD plus 3 doses of pentavalent DPT-Hib-HepB vaccine at 2, 3, and 4 months of age in 2010. With 67–94% coverage of HepB3 containing vaccine and 27–40% coverage of HepB-BD between 2007 and 2009^15^, the prevalence of HBsAg+children born in 2007–2008 fell to 1.6%^16^ from 12.5% among infants in pre-vaccine era^14^. Hepatitis B immune globulin (HBIG) at birth is not routinely recommended under the current strategy in Vietnam.

Risk factors associated with HBV infection among Vietnamese children were ethnicity other than Kinh, birth at home, older age group, no HepB, and missing HepB-BD^16^, while infants born to HBeAg+mothers with high HBV DNA titers, infants born to younger mothers, HBV DNA detectable in cord blood, inadequate or delayed HepB-BD, missing HBIG, and vaccine escape mutations of the HBV surface gene were reported as associated factors with immunoprophylactic failure in China and Lao PDR^17–20^.

Several cross-sectional surveys but few prospective studies have been conducted to identify the effectiveness of the current preventive measures against HBV infection of Vietnamese children so far. Thus, we conducted a prospective birth cohort study recruiting mothers and followed up their children at 2-year of age in central Vietnam, to determine the seroprevalence of hepatitis B virus in pregnant women and their children, and associated risk factors with HBV infection and anti-HBs antibody below the seroprotective level.

## Results

### Enrollment of subjects in the birth phase and their profiles

**Figure 1:**
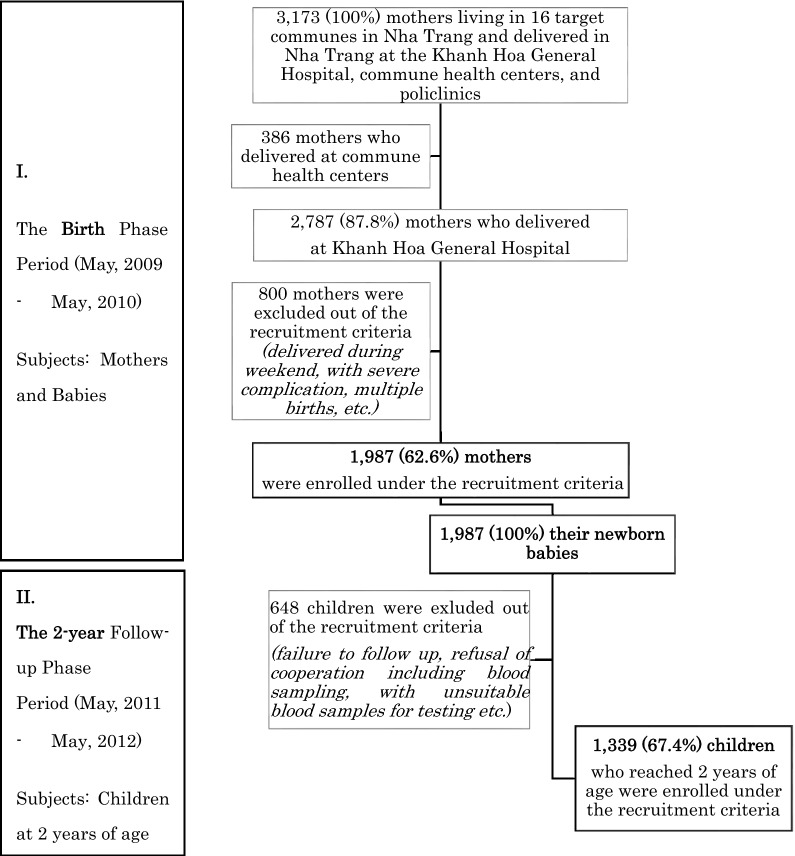
Study algorithm on enrollment of the subjects, Nha Trang, Vietnam, 2009–2012. This figure represents the study algorithm on enrollment of the subjects. I. During the birth phase period in May, 2009–May, 2010, a total of 3,173 mothers living in 16 target communes in Nha Trang delivered at either the Khanh Hoa General Hospital and commune health centers. Of them, 2,787 (87.8%) delivered at the hospital and 1,987 (62.6%) mothers and their 1,987 newborn babies were enrolled under the criteria. II. During the 2-year follow-up phase period in May, 2011–May, 2012, among these 1,987 neonates enrolled in the birth phase, 1,339 (67.4%) children who reached 2 years of age were enrolled under the criteria.

During the birth phase period (2009 May to 2010 May), a total of 3173 facility-based deliveries by mothers living in 16 communes of Nha Trang occurred at Government health facilities; Khanh Hoa General Hospital (KHGH), all commune health centers and polyclinics, based on delivery records in each facility, while births at home were considered to be negligible and no private facilities dealt with deliveries in Nha Trang during the study period. Of them, 2787 (87.8%) occurred at the hospital and 1987 (62.6%) mothers who fitted the recruitment criteria were enrolled at the birth phase of this study. The study algorithm on enrollment of the subjects is illustrated in Fig. 1.

The mean age of enrolled mothers was 27.9 years ranging from 17 to 45 years with a normal distribution. All mothers belonged to the Kinh ethnic group. 15.4% (305/1987) of mothers received antenatal care (ANC) less than 4 times, and 25.8% (512/1987) showed a low body mass index (BMI) in pre-pregnancy. 41.8% (831/1987) of deliveries were by Cesarean Section (CS), and the majority (92.4%, 768/831) of them were recorded as emergency CS being considered to be at a higher risk of mother-to-child transmission of HBV at birth in general. 48.4% (962/1987) of newborns were female, and 3.4% (68/1987) were preterm births (Table 1).

**Table 1:** Demographic characteristics and factors associated with HBV infection in mothers in the birth phase, Nha Trang, Vietnam, 2009–2010 (N = 1989).

Factors	Total N (%)	HBV infection in mothers n (% n/N)	OR (95% CI)	aOR (95% CI)
	N = 1989	n = 250 (12.6%)		
**【Mothers】**				
HBsAg				
Negative	1737 (87.4)	-	-	-
Positive	250 (12.6)	-	-	-
Anti-HBs antibody (mIU/ml)				
< 10	1033 (53.5)	226 (21.9)	Ref	Ref
≥ 10	898 (46.5)	18 (2.0)	0.1 (0.04, 0.1)	0.1 (0.04, 0.1)
Age (years old)				
17–24	572 (28.8)	61 (10.7)	Ref	Ref
25–34	1164 (58.6)	158 (13.6)	1.3 (0.96, 1.8)	1.5 (1.1, 2.1)
35–44	250 (12.6)	31 (12.4)	1.2 (0.7, 1.9)	1.5 (0.9, 2.6)
Maternal Education				
High school or higher	1060 (53.5)	110 (10.4)	Ref	Ref
None-Junior high school	920 (46.5)	139 (15.1)	1.5 (1.2–2.0)	1.5 (1.1, 2.0)
Residential Area				
Urban	1395 (70.2)	176 (12.6)	Ref	-
Suburban	592 (29.8)	74 (12.5)	1.0 (0.7, 1.3)	-
Para				
Multi	992 (49.9)	125 (12.6)	Ref	-
Primi	995 (50.1)	125 (12.6)	1.0 (0.8, 1.3)	-
Antenatal care				
≥ 4times	1682 (84.7)	201 (12.0)	Ref	Ref
< 4 times	305 (15.4)	49 (16.1)	1.4 (1.0, 2.0)	1.4 (0.96, 2.1)
BMI in prepregnancy				
Normal-High	1475 (74.2)	183 (12.4)	Ref	-
Low	512 (25.8)	67 (13.1)	1.1 (0.8, 1.4)	-
Anemia				
Negative	1478 (74.4)	191 (12.9)	Ref	-
Positive	509 (25.6)	59 (11.6)	0.9 (0.6, 1.2)	-
Mode of delivery				
Vaginal	1156 (58.2)	137 (11.9)	Ref	-
Caesarian Section	831 (41.8)	113 (13.6)	1.2 (0.9, 1.5)	-
Term of delivery				
Term-Postterm	1919 (96.6)	244 (12.7)	Ref	-
Preterm	68 (3.4)	6 (8.8)	0.7 (0.3, 1.6)	-

Logistic regression was used for projecting odds ratios (OR) in univariate analysis and adjusted odds ratios (aOR) in multivariate analysis.

Variables with statistical significance were underlined when 95%CI of aOR did not include 1.

HBsAg: hepatitis B surface antigen; HBV: hepatitis B virus; N: number; OR: odds ratio; CI: confidence interval; aOR: adjusted odds ratio; anti-HBs antibody: anti-hepatitis B surface antibody; HBeAg: hepatitis B e antigen; BMI: body mass index; and Ref.: reference.

### Serological status of mothers

Maternal HBsAg+rate was 12.6% (250/1987 [95% confidence interval (CI): 11.1–14.0], and 42.1% (98/233 [35.7–48.4]) out of 233 HBsAg+mothers were HBeAg+. No statistical difference in characteristics was detected between HBeAg+ and HBeAg− mothers. 56.9% (1130/1987 [54.7–59.0]) of mothers were either HBsAg-seroprotective or anti-HBs antibody at the seroprotective level. 122 mothers were randomly selected from the 233 HBsAg+mothers, and HBV DNA copy numbers were measured among them. The HBV DNA load ranged 4–9 Log IU/mL and the mean copy number was 5.5 Log IU/mL (95%CI: 5.1–5.8). Among HBsAg+mothers, mean HBV DNA copy numbers of 58 HBeAg+mothers were statistically higher than those of 57 HBeAg- mothers (6.7 (95%CI: 6.3–7.2) Log IU/mL, and 4.1 (95%CI: 4.0–4.3) Log IU/mL respectively; *p* value < 0.01).

### Factors associated with HBsAg positivity in mothers

Mothers with anti-HBs antibody below the seroprotective level (adjusted odds ratio [aOR] 0.1 [0.04–0.1] in those immune to HBV, compared to those with the protective level of antibody), mothers aged 25–34 years (aOR 1.5 [1.1–2.1], compared to those aged 17–24 years) and lower maternal education levels, *i.e.* none-junior high school (aOR 1.5 [1.1–2.0], compared to high school or higher level) were associated with HBV infection in mothers by logistic regression in multivariate analysis (Table 1). Prematurity was not associated with maternal HBV infection.

### Enrollment of subjects in the 2-year follow-up phase and their profiles

Among 1987 newborns enrolled in the birth phase, 1339 (67.4%) children were enrolled in the 2-year follow-up phase. The main reasons for lost to follow-up included failure to track (move-out) and lack of consent for blood sampling. The profiles of mothers in this group were similar to those in the birth phase (Table 3).

### Immunization and serological status of mothers and children in the follow-up phase

Among 1339 enrolled mothers, 166 were HBsAg+. 92.1% (1233/1339) of children received HepB3, and 82.5% (1104/1339) had HepB-BD within 24 h of birth. Those who received HepB3 including HepB-BD, i.e. complete HepB accounted for 76.5% (1024/1339), while 3 children did not receive any HepB. Among 166 children born to HBV-infected mothers, only 3 children received HBIG at birth.

The proportion of HBsAg-positivity in 2-year-old children was 1.9% (26/1339, 95%CI: 1.2–2.7). Twenty-five infected children were born to HBV-infected mothers and only one to a non-infected mother. 15.1% (25/166 [9.6–20.6] of children born to HBsAg+mothers got infected. Among children born to HBsAg+mothers, 33.8% (23/68) versus 0 (0/87) of children born to HBeAg+or HBeAg- mothers were infected (p < 0.001). Maternal HBeAg test results were obtained in 23 out of 26 infected children and all of them were HBeAg+. In contrast, the proportion of anti-HBs antibody below the seroprotective level in 2-year-old children was 15.2% (203/1338, 95%CI: 13.2–17.1). Among even at high-risk children as born to HBV-infected mothers, two-thirds (22/33) of children with anti-HBs antibody below the seroprotective level escaped HBV infection.

Of 166 children born to HBV-infected mothers, 122 received complete HepB doses and 44 received incomplete HepB doses. 13.1% (16/122 [7.0–19.2]) of those who received complete HepB doses and 20.5% (9/44 [8.0–32.9]) of those with incomplete HepB doses got infected. Furthermore, among children born to HBeAg+mothers, 28.3% (15/53 [15.8–40.8]) versus 53.3% (8/15 [24.7–81.9]) of children who received complete or incomplete HepB doses got infected. 14.3% (176/1232) of children showed anti-HBs antibody below the protective level despite receiving HepB3. Children born to mothers with higher HBV DNA copies in maternal blood at birth were more likely to get infected with HBV. Children born to mothers with 6–9 Log IU/mL of HBV DNA copy number were more likely to get infected compared to those born to mothers with 4 Log IU/mL defined as a reference, and the higher HBV DNA copy number mothers presented, the higher OR of HBV-infected children (Table 2). Mean HBV DNA copy number in mothers was statistically higher in HBV-infected children (OR 7.6 [7.1–8.1]) than in non-infected children (OR 4.9 [4.6–5.2]).

**Table 2:** Association of HBV infection in children with HBV DNA copy number in mothers in the 2-year follow-up phase, Nha Trang, Vietnam, 2011–2012 (N = 122).

HBV-DNA Copy number (Log IU/mL)	Total N (%)	HBV infection in children n (% n/N)	OR (95%CI)
	N = 122	n = 26	
4	71 (58.2)	1 (1.4)	Ref
5	2 (1.6)	0 (0)	*
6	7 (5.7)	3 (42.9)	52.5 (4.4–625.2)
7	15 (12.3)	7 (46.7)	61.3 (6.7–563.6)
8	18 (14.8)	9 (50.0)	70 (7.9–618.9)
9	9 (7.4)	6 (66.7)	140 (12.6–1561.7)
Total	122 (100)	26 (21.3)	-

Logistic regression was used for projecting odds ratios (OR) in univariate analysis.

Variables with statistical significance were underlined when 95%CI of OR did not include 1.

*Omitted due to lack of samples for analysis.

HBV: hepatitis B virus; DNA: deoxyribonucleic acid; HBsAg: hepatitis B surface antigen; N: number; OR: odds ratio; CI: confidence interval; and Ref.: reference.

Among 3 children who received HBIG at birth, 2 children born to HBeAg+mothers had 7–8 Log IU/mL of HBV DNA titer: one with complete HepB doses escaped from infection and the other with incomplete HepB doses got infected. The third child was born to an HBeAg- mother and did not get infected despite incomplete HepB doses.

### Factors associated with HBV infection and anti-HBs antibody below the seroprotective level in children

Maternal HBV infection (aOR 217.4 [28.6–1651.5]), low BMI in pre-pregnancy (aOR 4.9 [1.9–12.6], compared to normal-high BMI), and anti-HBs antibody below the seroprotective level in children (aOR 0.2 [0.1–0.7] at 10–99 mIU/mL and aOR 0.1 [0.04–0.4] at 100 mIU/mL or higher, compared to < 10 mIU/mL) were associated with HBV infection of children by logistic regression in multivariate analysis (Table 3).

**Table 3:** Demographic characteristics and factors associated with HBV infection in the 2-year follow-up phase, Nha Trang, Vietnam, 2011–2012 (N = 1339).

Factors	Total N (%)	HBV infection in children n (% n/N)	OR (95%CI)	aOR (95%CI)
	N = 1339	n = 26 (1.9%)		
**[Children]**				
HBsAg				
Negative	1313 (98.1)			
Positive	26 (1.9)			
Anti-HBs antibody (mIU/ml)				
< 10	203 (15.2)	12 (5.9)	Ref	Ref
10–99	615 (46.0)	9 (1.5)	0.2 (0.1, 0.6)	0.2 (0.1, 0.7)
≥ 100	520 (38.9)	5 (1.0)	0.2 (0.1, 0.4)	0.1 (0.04, 0.4)
Sex				
Female	639 (47.7)	15 (2.4)	Ref	-
Male	700 (52.3)	11 (1.6)	1.5 (0.7, 3.3)	-
Immunization status of HepB				
No or Incomplete	315 (23.5)	10 (3.2)	Ref	
Complete	1024 (76.5)	16 (1.6)	0.5 (0.2–1.1)	-
Timing of HepB-BD				
No	229 (17.1)	6 (2.6)	Ref	-
Day 0–1	1104 (82.5)	20 (1.8)	0.7 (0.3, 1.7)	-
Day 2–7	6 (0.5)	0 (0)	*	-
HBIG at birth				
No	1333 (99.8)	25 (1.9)	Ref	Ref
Yes	3 (0.2)	1 (33.3)	26.2 (2.3, 298.0)	10.3 (0.8, 133.9)
**[Mothers]**				
HBsAg				
Negative	1173 (87.6)	1 (0.1)	Ref	Ref
Positive	166 (12.4)	25 (15.1)	207.8 (27.9, 1545.3)	217.4 (28.6, 1651.5)
Age (years old)				
17–24	385 (28.9)	11 (2.9)	Ref	-
25–34	769 (57.8)	14 (1.8)	0.6 (0.3, 1.4)	-
35–44	177 (13.3)	1 (0.6)	0.2 (0.02, 1.5)	-
BMI in prepregnancy				
Normal-High	997 (74.5)	12 (1.2)	Ref	Ref
Low	342 (25.5)	14 (4.1)	3.5 (1.6, 7.7)	4.9 (1.9, 12.6)
Mode of delivery				
Vaginal	766 (57.2)	18 (2.4)	Ref	-
Caesarian Section	573 (42.8)	8 (1.4)	0.6 (0.3, 1.4)	-

Logistic regression was used for projecting odds ratios (OR) in univariate analysis and adjusted odds ratios (aOR) in multivariate analysis.

Variables with statistical significance were underlined where 95%CI of aOR did not include 1.

HBsAg: hepatitis B surface antigen; HBV: hepatitis B virus; anti-HBs antibody: anti-hepatitis B surface antibody; N: number; OR: odds ratio; CI: confidence interval; aOR: adjusted odds ratio; HepB: hepatitis B vaccine; HepB-BD: birth dose of HepB; incl.; including; w/o: without; HBIG: hepatitis B immune globulin; BMI: body mass index; and Ref.: reference.

*Dropped since no HBsAg-positive cases existed in the category.

For those born to HBV-infected mothers, irrespective of immunization status, there was no statistical difference in HBV infection in children between those born by CS and vaginal delivery, or between emergency and elective CS.

Meanwhile, HBV infection in children (aOR 5.5 [2.4–12.2], compared to non-infection in children), maternal age of 35–44 years (aOR 1.9 [1.2–3.1], compared to 17–24 years), boys (aOR 1.5 [1.1–2.0], compared to girls), and no or incomplete doses of HepB (aOR 0.7 [0.5–0.9] incomplete HepB doses, compared to no or incomplete doses of HepB) were associated with anti-HBs antibody below the seroprotective level in children by logistic regression in multivariate analysis (Table 4).

**Table 4:** Demographic characteristics and factors associated with anti-HBs antibody below the seroprotective level in children in the 2-year follow-up phase, Nha Trang, Vietnam, 2011–2012 (N = 1338).

Factors	Total N (%)	Anti-HBs antibody below the seroprotective level n (% n/N)	OR (95%CI)	aOR (95%CI)
	N = 1338	n = 203 (15.2%)		
**[Children]**				
HBsAg				
Negative	1313 (98.1)	191 (14.6)	Ref	Ref
Positive	26 (1.9)	12 (46.2)	5.0 (2.3, 11.0)	5.5 (2.4, 12.2)
Sex				
Female	639 (47.7)	81 (12.7)	Ref	Ref
Male	700 (52.3)	122 (17.5)	1.5 (1.1, 2.0)	1.5(1.1, 2.0)
Immunization status of HepB				
No or Incomplete	315 (23.5)	62 (19.7)	Ref	Ref
Complete	1024 (76.5)	141 (13.8)	0.7 (0.5–0.9)	0.7 (0.5–0.9)
Timing of HepB-BD				
No	229 (17.1)	39 (17.0)	Ref	-
Day 0–1	1104 (82.5)	163 (14.8)	0.8 (0.6, 1.2)	-
Day 2–7	6 (0.5)	1 (16.7)	1.0 (0.1, 8.6)	-
HBIG at birth				
No	1333 (99.8)	202 (15.2)	Ref	-
Yes	3 (0.2)	0 (0)	*	-
**[Mothers]**				
HBsAg				
Negative	1173 (87.6)	170 (14.5)	Ref	-
Positive	166 (12.4)	33 (19.9)	1.5 (0.97, 2.2)	-
Age (years old)				
17–24	385 (28.9)	50 (13.0)	Ref	Ref
25–34	769 (57.8)	115 (15.0)	1.2 (0.8, 1.7)	1.2 (0.9, 1.8)
35–44	177 (13.3)	37 (21.0)	1.8 (1.1, 2.9)	1.9 (1.2, 3.1)
BMI in prepregnancy				
Normal-High	997 (74.5)	150 (15.1)	Ref	-
Low	342 (25.5)	53 (15.5)	1.0 (0.7, 1.5)	-
Mode of delivery				
Vaginal	766 (57.2)	124 (16.2)	Ref	-
Caesarian Section	573 (42.8)	79 (13.8)	0.8 (0.6, 1.1)	-

Logistic regression was used for projecting odds ratios (OR) in univariate analysis and adjusted odds ratios (aOR) in multivariate analysis.

Variables with statistical significance were underlined where 95%CI of aOR did not include 1.

HepB: hepatitis B vaccine; HBsAg: hepatitis B surface antigen; N: number; OR: odds ratio; CI: confidence interval; aOR: adjusted odds ratio; HepB-BD: birth dose of HepB; HBIG: hepatitis B immune globulin; HLA: human leukocyte antigen; BMI: body mass index; and Ref.: reference.

*Dropped since no HBsAg positive cases existed in the category.

## Discussion

This hospital-based prospective cohort study demonstrated the seroepidemiological features, risk factors associated with HBV infection, and immunity to it in children, providing implications for the current preventive strategy.

This study demonstrated that 12.6% [11.1–14.0] of mothers were HBsAg+, and 42.1% of HBsAg+mothers were HBeAg-. These rates were slightly higher than the previous findings in Vietnam; 9.5% of pregnant women were HBsAg+^10^, and 16.4–38.2% of adult carriers were HBeAg+^12–14^.

In this study, anti-HBs antibody below the seroprotective level, older ages, and lower educational levels in mothers were associated with current HBV infection of mothers which was in good agreement with the previous studies^12,13^.

Despite the high maternal HBV carrier rate (12.6%), the overall proportion of HBV infection among 2-year-old children was found to be as relatively low as 1.9% [1.2–2.7] with over 90% of children receiving HepB3 and more than three quarters having complete HepB doses. This prevalence corresponds to the one from a previous cross-sectional study reporting HBsAg-positivity rate to be 1.6% among children born in 2007–2008 in Vietnam^16^. Such a level of risk reduction was expected by the previous study projecting 84.8–89.7% of risk reduction in children in reference to 12.5–18.4% in the pre-vaccination era^14^. This was similar to the one in Lao PDR (1.7%) but higher than in Cambodia (0.6%), China (0.3%), and Thailand (0.3%), and lower than in Myanmar (3.8%) in 2012–2017^21,22^. Thus, a successful universal HepB vaccination program could significantly avert perinatal and infantile HBV infection in central Vietnam despite a high-risk condition of vertical and horizontal HBV infection from adult carriers born in the pre-vaccine era.

Our study indicated that 15.2% of children at 2 years of age had anti-HBs antibody below the seroprotective level, which was consistent with other studies suggesting it to be 12.5–20%^23,24^. Such status may mainly include either primary vaccine failure with complete or incomplete HepB, or waning anti-HBs antibody below the protective level by the age. Two-thirds (22/33) of children born to HBsAg+mothers with anti-HBs antibody below the seroprotective level escaped HBV infection possibly due to memory immune to HBV.

HBV infection in mothers, anti-HBs antibody below the seroprotective level in children, and mothers with low BMI in pre-pregnancy were associated with HBsAg-positivity in children. Children born to mothers with higher HBV DNA copy number were more likely to get infected with HBV as previously observed^17–20^. Obesity may decrease response to HepB^25,26^, no study has found an association of low BMI in prepregnancy with HBV infection in their children. This may be linked to lower socioeconomic disadvantage and health-seeking behavior including immunization. Association between chronic HBV infection and preterm birth were controversial in previous studies^27,28^ and our study revealed no association between them. There is insufficient evidence that CS reduce the risk of mother-to-child transmission of HBV after immunoprophylaxis^29,30^. and our study was in agreement with it. Unlike the previous study^16^, immunization status was not indicated to be statistically associated with HBV infection in children in this study, partly due to the small sample size.

Our study revealed that as much as 28.3% of children born to HBeAg+mothers have got infected even with complete HepB. To prevent mother-to-child transmission of HBV from high-risk mothers further, HBIG at birth would be an option, which is rarely offered only in the private sector mostly in big cities at a high cost. However, some have reported its insufficient efficacy in very high-risk cases. Since only 3 children received it in this study, its efficacy was unable to be evaluated. Instead, anti-viral therapy in pregnant women at high risk has indicated promising evidence on its efficacy and safety^31^ as recommended by international liver diseases associations and World Health Organization (WHO)^32–34^.

HBV infection in children, older maternal age, no or incomplete doses of HepB, and boys were associated with anti-HBs antibody below the seroprotective level in children in this study. We found that older maternal age was associated with anti-HBs antibody below the seroprotective level in children which was contradictory to a previous finding^20^. High seroprevalence of HBV among women of childbearing age with a slow introduction of the nationwide HepB program may have influenced it. Males responded less to HepB in this study as reported previously^35–37^. Moreover, no or incomplete doses of HepB was associated with anti-HBs antibody below the seroprotective level in children, while an association of delayed HepB-BD with immunoprophylactic failure as reported previously^18^ was not observed in this study.

This study has some limitations. Firstly, approximately 10% of mothers who delivered at either health centers or home and their babies, were not enrolled which could bring some selection bias. Such group of people with restricted medical resources and limited health consciousness are more likely to miss the opportunity of receiving appropriate perinatal and infantile practices including HepB. Thus, the prevalence overall of HBV infection among children could be underestimated, therefore we should be cautious in generalizing the findings. Secondly, due to the logistic and feasibilities issues we were only able to conduct a follow-up survey at 2 years of age, although one to three months after the last dose of HepB would be more appropriate timing for the evaluation. This would have increased the rate of failure to follow-up and the probability of horizontal infection from family or others. We also did not collect the information on possible risk factors linked to horizontal infection such as family history of HBV. These conditions would have made interpretation of the findings difficult. Finally, the immune status induced by HepB might be underestimated since the anti-HBs antibody was measured at 2 years of age when the titers may have waned. Nonetheless, some seroprotective effect was projected among children due to memory immune to HBV even with anti-HBs antibody below the seroprotective level as shown previously.

## Conclusions

Our birth cohort study determined a low rate of congenital HBV infection and associated risk factors in Vietnam. Further studies are needed to advance prevention including anti-viral therapy in pregnant women at high risk.

## Methods

### Study design, period, and location

A hospital-based birth cohort study was conducted at KHGH in Nha Trang with a population of approximately 390,000 that is the capital city of Khanh Hoa Province, central Vietnam from May 2009 to May 2010 as the birth phase and from May 2011 to May 2012 as the 2-year follow-up phase. KHGH is a provincial hospital with 900 beds which provides comprehensive medical services including approximately 6000 deliveries annually. The detail of this hospital was described previously^38^.

### HepB vaccination schedule under the national immunization program

The national immunization program for HepB vaccination was revised in 2010 from monovalent HepB-BD followed by 2 times of monovalent HepB at 2 and 4 months of age to monovalent HepB-BD followed by 3 times of pentavalent DPT-Hib-HepB at 2, 3, and 4 months of age. The revised program was introduced in June 2010 to KHGH. During the study period, Gene-HBvax (Vabiotech company, Vietnam) was used as a monovalent HepB for HepB-BD, and Quinvaxem (Berna Biotech company, Korea) was used as pentavalent HepB for further HepB doses at KHGH, while vaccines used in health facilities outside the hospital were unknown.

### Subjects and recruitment process

The study targeted residents in 16 out of 27 communes/wards in Nha Trang. Nha Trang is a tourist city, and 16 residential communes/wards were selected as the target area for this study while the remaining 11 communes/wards where big blocks of government administrative buildings, hotels, and restaurants were mostly located were excluded.

In the birth phase, we recruited mothers aged 17 years or older residing in the above areas in Nha Trang who delivered a single child at the hospital on weekdays during this study period without severe maternal complications together with their newborn babies. After we obtained written informed consent from mothers, we collected demographic, clinical, and epidemiological information including self-reported pre-pregnancy body weight through interviews using a structured questionnaire before births as well as from medical charts or maternal health cards. Maternal blood was also collected before deliveries. Clinical status on newborns was examined and recorded after birth. The whole process for collecting information and samples was performed by two trained research nurses under the supervision of a research pediatrician.

In the 2-year follow-up phase, we requested the children recruited in the birth phase to visit their local commune health centers on a designated day within 2 weeks before or after their 2-year birthday. We enrolled those whose caregivers agreed on the condition for further survey including blood sampling. After informed consents were obtained, epidemiological information including the history of immunization was collected through interview using a structured questionnaire and from immunization card. Clinical examination and blood sampling were then conducted and recorded. Health staffs in 16 commune health centers in this study area were carefully trained to perform the prescribed procedures every two weeks, and two trained research staff from Khanh Hoa Provincial Health Service Department supervised them in each center throughout the study period. Vaccination histories were validated through records at commune health centers concurrently.

### Sample collection and testing

In the birth phase, plasma samples were separated from maternal blood by centrifugation and kept at − 80 ℃ freezers at the hospital. The samples were then transported to Nagasaki, Japan, where HBsAg and anti-HBs antibody for all, and HBeAg only for HBsAg+cases were examined by chemiluminescent immunoassay (CLIA) (ARCHITECT HBsAg-QT, AUSAB, and HBeAg, Abbott Japan, Tokyo, Japan, respectively). The cut-off values of HBsAg, anti-HBs antibody, and HBeAg were 0.05 International Unit (IU)/mL, 10.0 mIU/mL, and 1.00 S/CO (sample mean chemiluminescent signals [RLUs] /cut-off RLUs), respectively, following the manufacturer’s guidelines. HBV DNA titers in mothers were detected by transcription-mediated amplification (TMA) methods in 3.7–8.7 logarithm genome equivalent (LGE)/ml (DNA probe [FR]-HBV, Fujirebio, Tokyo, Japan).

In the 2-year follow-up phase, blood samples from children were processed similarly as above at Pasteur Institute in Nha Trang and then transported to Nagasaki University, Japan where HBsAg was tested by CLIA (ARCHITECT HBsAg-QT, Abbott Japan, Tokyo, Japan) with 0.05 IU/mL of the cut-off value. Anti-HBs antibody was measured by an assay developed by Green Peptide Co. Ltd., Kurume, Japan using MAGPIX Bead Array System (Luminex, Austin, Texas, USA) with 10 mIU/mL of the cut-off value.

### Characteristic definition and categorization

Maternal age was grouped into 17–24, 25–34, or 35–44 years old. Maternal educational level was grouped into low *i.e.* none or up to junior high school (0–9 years of schooling) and high *i.e.* high school or higher (10 years of schooling or more). Maternal residential areas were grouped into suburban and urban, defined by a municipal document according to the communes. The numbers of mothers who received ANC 4 times or more, as recommended by WHO^39^, and those less than 4 times were calculated. BMI was defined as the weight in kilograms divided by the square of the height in meters (kg/m^2^), and underweight was defined as BMI less than 18.50 according to the WHO^40^. Anemia for pregnant women was defined as less than 11 g/dl of haemoglobin^41^, and the preterm is determined as neonates born alive before 37 weeks of gestation according to the WHO^42^.

The protective concentration of anti-HBs antibody was determined as ≥ 10 mIU/mL as indicated by WHO^1^. Anti-HBs antibody levels were categorized into less than 10 mIU/mL (negative or nonimmune to HBV), 10–99 mIU/mL (positive or immune to HBV), or 100 mIU/mL or higher (highly positive or immune to HBV) in this study. HepB-BD was defined as a birth dose of monovalent HepB received within 7 days after birth for this study, and categorized by the timing of receipt into either within 24 h or 2–7 days of life. HepB3 was defined as immunization of a total of 3 or more doses of either monovalent or pentavalent HepB. Immunization status was categorized into complete HepB doses (defined as HepB3 including HepB-BD) or incomplete HepB doses (defined as HepB3 without HepB-BD, or less than 3 doses of HepB with or without HepB-BD). Nonresponse to HepB3 was defined as negative or nonimmune to HBV despite HepB3 given.

### Data management and statistical analysis

All the collected information was managed confidentially throughout the process. The data were double-entered and cleaned, and statistical analysis was conducted with STATA 11.1. The study algorithm on enrollment of the subjects was illustrated.

An association of HBV DNA copy number in mothers with HBV infection in their children was analyzed using logistic regression, and tabulated in numbers, proportions, and OR with 95%CI. Here, we used the lowest copy number 4 Log IU/ml as a reference to calculate the OR of HBV infection in subjects with higher copy numbers. Mean HBV DNA copy numbers among mothers with HBV-infected and -non-infected children were compared and analyzed using t-test, and ORs with 95%CI were described.

Main factors in demographic profiles, clinical-epidemiological factors including seroprevalence, and immunization status were tabulated in numbers and proportions. HBV infection in children was profiled and figured by maternal serological features, and the immunization status of HepB in children, while mean maternal HBV DNA titer was analyzed by HBV infection and immunization status of HepB in children. HBV-infection status with immunization status of HepB in children who received HBIG was described.

Factors associated with HBV infection in mothers, HBV infection in children, or non-response to HepB3 in children were analyzed by univariate analysis respectively using logistic regression. Exposure variables for mothers included HBsAg-positivity, at the seroprotective level of anti-HBs antibody, HBeAg-positivity, age group, ethnicity, educational level, marital status, residential area, previous pregnancy (gravida) and delivery (parity), ANC visit, BMI in prepregnancy, anemia, mode and term of delivery for mothers. Exposure variables for children included HBsAg-positivity, anti-HBs antibody level, sex, low birth weight, small-for-gestational-age, the timing of HepB-BD, immunization status of HepB, and receipt of HBIG at birth. Of these, the main findings were tabulated with OR and their 95%CI. Factors were defined to be associated with HBV infection in children with statistical significance when the 95%CI of OR did not include 1.0. Associated factors were included as exposure variables for further multivariate analysis using logistic regression, being represented with aOR and their 95%CI.

### Ethics declarations

This study was approved by the Ethical Committees of Nagasaki University, Japan (# approval no.160908158), and the National Institute of Hygiene and Epidemiology, Vietnam. Written informed consent was obtained from all participants, and all data were kept confidentially and anonymously throughout the process. All methods were carried out in accordance with relevant guidelines and regulations. The informed consent from a parent and/or legal guardian of the participants as minors (mothers who are under 18) are involved in the study.

## Data Availability

The datasets generated during and/or analyzed during the current study are available from the corresponding author on reasonable request.
